# Recent advances in acupuncture for pain relief

**DOI:** 10.1097/PR9.0000000000001188

**Published:** 2024-09-13

**Authors:** Supranee Niruthisard, Qiufu Ma, Vitaly Napadow

**Affiliations:** a Pain Management Research Unit, Department of Anesthesiology, Faculty of Medicine, Chulalongkorn University and King Chulalongkorn Memorial Hospital, Bangkok, Thailand; b Center of Bioelectronic Medicine, School of Life Sciences, Westlake University, Hangzhou, China; c Department of Physical Medicine and Rehabilitation, Spaulding Rehabilitation Hospital, Harvard Medical School, Charlestown, MA, USA; d Athinoula A. Martinos Center for Biomedical Imaging, Massachusetts General Hospital, Harvard Medical School, Charlestown, MA, USA

**Keywords:** Acupuncture, Pain management, Inflammation, Integrative pain care, Nonpharmacological approach for pain

## Abstract

**Introduction:**

Acupuncture therapy has achieved global expansion and shown promise for health promotion and treatment of acute/chronic pain.

**Objectives:**

To present an update on the existing evidence base for research and clinical practice supporting acupuncture analgesia.

**Methods:**

This Clinical Update elaborates on the 2023 International Association for the Study of Pain Global Year for Integrative Pain Care “Factsheet Acupuncture for Pain Relief” and reviews best evidence and practice.

**Results:**

Acupuncture is supported by a large research evidence base and growing utilization. Mechanisms of acupuncture analgesia include local physiological response at the needling site, suppression of nociceptive signaling at spinal and supraspinal levels, and peripheral/central release of endogenous opioids and other biochemical mediators. Acupuncture also produces pain relief by modulating specific brain networks, integral for sensory, affective, and cognitive processing, as demonstrated by neuroimaging research. Importantly, acupuncture does not just manage pain symptoms but may target the sources that drive pain, such as inflammation, partially by modulating autonomic pathways. Contextual factors are important for acupuncture analgesia, which is a complex multifaceted intervention. In clinical practice, historical records and many providers believe that acupuncture efficacy depends on specific acupoints used, the technique of needle placement and stimulation, and the person who delivers the procedure. Clinical research has supported the safety and effectiveness of acupuncture for various pain disorders, including acupuncture as a complementary/integrative therapy with other pain interventions.

**Conclusion:**

Although the quality of supportive evidence is heterogeneous, acupuncture's potential cost-effectiveness and low risk profile under standardized techniques suggest consideration as a neuromodulatory and practical nonpharmacological pain therapy.

## Abstract 2

With the heterogeneity of supportive evidence, acupuncture's low-risk profile under standardized techniques and potential cost-effectiveness suggest consideration as a neuromodulation/practical nonpharmacological management of pain therapy.

Supplemental Digital Content is Available in the Text.

**Key Points**

1. Acupuncture has been one of the most used nonpharmacological modalities for integrative pain care as monotherapy or a complement of other techniques with increasing supportive evidence not only by basic research in animals but also by translational and clinical research.
2. Safe acupuncture procedures for pain control with minimal adverse effects are provided by licensed acupuncturists and trained medical doctors.
3. The effectiveness of acupuncture depends on many factors, including the specific acupoints used, the technique of needle placement and stimulation, and the person who provides the procedure.
4. Understanding the science and mechanisms of action underlying acupuncture analgesia helps improve the effectiveness of the treatment of different pain conditions. Acupuncture produces pain relief by modulating multiple brain networks, integral for sensory, affective, and cognitive processing and by modulating the sources that drive pain such as inflammation.

## 1. Introduction

Pain is a complex experience, modulated by many biopsychosocial interactions, making it difficult to be effectively treated by any single intervention. Recognizing the importance of combining healthcare strategies for successful pain care, International Association for the Study of Pain announced the 2023 Global Year of “Integrative Pain Care” and launched 12 related factsheets, 25 Global Year videos, and 2 webinars (Supplement 1, http://links.lww.com/PR9/A244). The term “Integrative Pain Care” is defined as temporally coordinated, mechanism-guided, individualized, and evidence-based integration of multiple pain treatment interventions.^4^ Among complimentary health approaches specifically for managing pain, acupuncture has been one of the most commonly used nonpharmacological modalities for effective pain care,^1,27,83^ and usage was found to increase after insurance coverage,^58^ suggesting that financial barriers have historically limited untapped demand. A recent publication in JAMA by the National Institute of Health found that the use of acupuncture, which was increasingly covered by insurance, increased from 1.0% in 2002 to 1.5% in 2012 and 2.2% in 2022 in the United States.^58^ Although acupuncture originated in Eastern Asia more than 2,500 years ago, the use of acupuncture as a therapy has now spread globally to promote health and treat multiple clinical disorders, including pain.^7,83^ Interestingly, when applied for the management of pain, acupuncture can not only control both acute pain^62^ and chronic pain^78^ but also address potential underlying pathophysiology, such as the reduction of inflammation.^64,76^

### 1.1. Real-world practice of acupuncture treatment

#### 1.1.1. Traditional needle acupuncture

The term “Traditional Needle Acupuncture (TNA)” in this Clinical Update is defined as the practice of acupuncture following the principles of Traditional East Asian Medicine, using filiform fine, sterile needles inserted through the skin at specific points identified on the body surface called “acupoints”. Acupuncture needles are shafts of solid metal (eg, stainless steel alloy), not hollow bore (as nothing is injected), and are typically disposable, single-use only, to reduce infection risk. In some resource-limited locations in the world, reusable acupuncture needles are sterilized with standard sterilization techniques, for example, steam under pressure or dry heat.^22,77^ Disinfecting reusable needles with alcohol is inadequate and has been reported to cause infection.^99^ The main philosophical components of Traditional East Asian Medicine, particularly those originated from China, involve the theory of meridians/acupoints and energy (sometimes called “Qi”) flow, Yin and Yang theory, 5 elements theory, which guide diagnosis and treatment decisions.^84^ Meridians, or channels, in Traditional East Asian Medicine are defined as energetic pathways located throughout the entire body that form a connection between discrete acupoints. Acupoints are located at specific sites throughout the human body, many belonging to 12 bilateral meridians and 2 midline meridians.^45,85^ Following diagnosis based on these principles,^83^ needling locations and stimulation technique are chosen by a trained practitioner, which is performed manually or in conjunction with electrical (electroacupuncture) or heat (thermal) stimulation. We should note that the historical *philosophy* underlying these principles has evolved over many years, following iteration and recorded empirical evidence, whose clinical outcomes may link to both physiological and psychological responses to acupuncture needling.

##### 1.1.1.1. Manual stimulation

Originally, acupuncture needle stimulation involved thrusting and lifting or rotating the needle until a sense of dull ache, numbness, fullness, or heaviness called “deqi” is achieved in the patient and/or needle grasp perceived by the acupuncturist, which is believed to correlate with better therapeutic effect.^23,31,60,95^ However, manual stimulation may be time consuming, and prolonged stimulation over the entire duration of treatment may not be feasible in general clinical practice because of time constraints.

##### 1.1.1.2. Electrical stimulation

Starting in the 1950s, electroacupuncture has been applied to augment manual stimulation by connecting metallic needles to an electrical stimulator, which supplies either high- or low-frequency impulses, or in combination, including adjustable voltage and waveform, allowing for stimulation to continue over a longer duration of treatment.^75^ The mechanisms of electroacupuncture analgesia involve local, peripheral, and central analgesic effects (see below). Recent research reveals that recruitment of neutrophils and releasing of β-endorphins at the inflammation site is a mechanism of local analgesia by electroacupuncture.^67^ The decision to include electroacupuncture is based on many factors, such as the pain conditions treated, patient's preference, and practitioner's training and preference.

##### 1.1.1.3. Thermal stimulation

Other forms of needle stimulation include thermal stimulation of acupuncture needles (ie, “moxibustion”), which involves slow burning moxa or dried mugwort leaves (Artemisia argyi), attached to inserted needles at the acupoints.^83^ According to Traditional East Asian Medicine, moxibustion can improve health by balancing “Qi.”^12,84^ Modern research suggests that mechanisms of moxibustion mainly relate to conductive and radiative heating, and pharmacological effects of burning moxa.^12^ However, basic research to fully understand the mechanisms of moxibustion is still in its infancy.

##### 1.1.1.4. Selection of points and needle stimulation

Acupoints selected to treat pain with TNA have been described in the traditional literature and include points local to the pain area in addition to acupoints considered appropriate for individual patients, personalized to their presentation.^33,96^ Very few clinical trials have directly compared the effects of manual, electrical, and thermal stimulation. However, studies comparing electroacupuncture and manual stimulation suggest that electroacupuncture elicits better control than manual stimulation for certain forms of pain.^39,40^ This difference could be due to differential responses of tissues underlying the acupoint, including nerves, to mechanical vs electrical stimulation, and/or due to differential duration of active stimulation because manual stimulation only occurs transiently during the treatment while electrical stimulation occurs throughout. However, such research on acupuncture stimulation needs to balance internal and external validity because, for example, it may be impractical to stimulate multiple needles manually for extended periods.

##### 1.1.1.5. Frequency and duration of acupuncture treatment

The benefit of acupuncture treatment not only depends on the appropriate selection of acupoints and stimulation techniques but also on the treatment session and treatment duration. For instance, acupuncture treatment for chronic pain of knee osteoarthritis 3 times a week resulted in better pain relief than once a week.^46^ The duration of treatment at least 5 weeks was found to achieve 80% maximum analgesic effect in neck pain, shoulder pain, and knee pain.^41^ The frequency and duration of adequate dose of acupuncture treatment vary in clinical practice depending on specific pain conditions and patient conditions, which have long been neglected to examine in the clinical trial studies.^50^ This is an important knowledge gap for future research.

#### 1.1.2. Western medical acupuncture

Western medical acupuncture (WMA) is the adaptation of traditional acupuncture to the biomedical ontology and is practiced by trained Western healthcare professionals. Western medical acupuncture is based on our modern understanding of anatomy, physiology, and pathology, and the principles of evidence-based medicine.^81^ Although the practice of WMA uses TNA concepts of acupoints, it does not involve the traditional philosophical constructs, such as Yin/Yang and circulation of “Qi.” Rather, WMA combines the needling of acupoints with principles of evidence-based medicine and clinical experience. Physical examination and diagnosis follow Western medicine to confirm that symptoms are amenable to acupuncture treatment as monotherapy or by integrating acupuncture with other interventions. Because acupoint nomenclature has been better standardized recently,^45,84,85^ it is generally used for communication among Western medical acupuncturists due to convenience. In terms of treatment techniques, there are relatively few differences between TNA and WMA. Both manual and electrical stimulation of needles are used in WMA. In regard to application, WMA is mainly focused on treating musculoskeletal pain, including myofascial trigger point pain. Prior research has suggested a high degree of correspondence (about 71%) between acupoints and trigger points,^57^ and inserting needles into such trigger points may elicit effects similar to dry needling (a WMA technique used to treat dysfunction of skeletal muscle and connective tissue).^6,16^ Similarity is also noted in needle response—eg, “deqi” sensation according to TNA may approximate a muscle twitch response targeted by WMA.^27^

## 2. Adverse effects of acupuncture treatment

Although acupuncture has been found to have an excellent safety record when performed by experienced, licensed practitioners, caution must be considered for patients with bleeding disorders,^28^ pacemaker devices (for electroacupuncture),^71^ or who are pregnant.^13,63,69,94^ Although pregnancy is considered a contraindication due to concern that acupuncture might affect hormone levels and nervous systems affecting uterine function, no evidence of adverse effects of acupuncture related to pregnancy causing serious observable maternal, fetal, or neonatal complications has been found.^13,63,69,94^ Side effects of acupuncture needling are usually mild and transient. The 3 most common reported adverse events were local pain from needling, skin flare around the needle insertion point,^65^ and slight bleeding or bruising,^3,88,92^ with the former 2 possibly regarded as expected responses to needling and even possibly therapeutically intended reactions.^65,105^ Rarely, patients may experience vasovagal effects, such as bradycardia or fainting. Some patients report clinical/physiological effects of acupuncture for a few hours (or even a day) after the treatment. These effects include feeling more relaxed, sleepy, fatigued, and improvement of sleep. Serious complications have been reported related to improper needling techniques and include infection and injury to internal organs or the nervous system.^92,99^ Pneumothorax is the most frequently reported serious acupuncture-related adverse event.^3^

## 3. Education/training and barriers of acupuncture treatment for pain relief

Several countries offer acupuncture training courses and degrees in TNA or WMA. More and more governments are adopting official licensing systems for acupuncture practitioners which require extensive accredited academic training, passing examinations, continuing medical education, and registration as licensed acupuncturists or certified acupuncture practitioners.^82^ Practitioners can also be trained to provide acupuncture treatment for pain control^22,82,86^ as monotherapy or as a complement to other treatments. The duration of the training depends on the individual program and local regulations. For instance, in the United States, there are 33 states which allow non-additional training physicians to provide acupuncture treatment, 11 states and the District of Columbia require 200 to 300 training hours and 3 states require physicians only to be licensed acupuncturists. The other states have variations of requirements range from no regulatory agency ruling to allowing non-healthcare professionals to be trained or allowing individuals without a clinical professional license to be trained as Acupuncture Detoxification Specialists.^5^ The barrier to produce sufficient number of acupuncturists is the different state-level or local training requirements; a model to increase access to acupuncture may be by allowing nonphysician medical professionals to complete reduced hours of training requirement for specific indications.^5^

Scientific research on acupuncture dates back many decades with fast growing of advanced ongoing research, which supports both clinical and mechanistic effects of acupuncture therapy for pain and inflammation. Although the most common indications of acupuncture practice related to chronic pain are low back pain, headache, arthritis, insomnia, neck pain, and frozen shoulder,^79^ increasing the evidence base for acupuncture and pain control will help guide future guideline development for nonpharmacological approaches to pain management. Barriers to implementing acupuncture into clinical practice include historical misconceptions related to physicians' knowledge/attitudes of this ancient therapy, patient preferences, and external factors, such as administrative policies/constraints, lack of resources, and insurance coverage. A recent study of the cost-effectiveness of acupuncture for pain relief in acute nonspecific low back pain showed cost-effectiveness from a 1-year perspective,^68^ whereas it was uncertain for chronic nonspecific low back pain.^72,74^ Studies of acupuncture in other pain conditions, which include measures of quality of life, have also shown promise to be cost-effective^25,61,87,89,90^ but must be extended across different healthcare systems internationally.

## 4. Basic science research

Scientists have continued to unravel the neuroanatomical basis of acupuncture in the past decade.^47,49^ For instance, mechanisms of acupuncture analgesia through direct modulation of pain transmission pathways include (1) local analgesic effects on both inflammatory and neuropathic pain in mice through the release of adenosine at the site of needling,^20^ (2) segmental spinal inhibition by low-intensity stimulation according to the gate control theory of pain and supraspinal inhibition by high-intensity stimulation according to diffuse noxious inhibitory controls,^101^ often studies in healthy animals or humans, and (3) peripheral and/or central release of endogenous opioid peptides and other chemical mediators.^9,10,23,51,56,97,103^ A notable caveat in these lines of acupuncture basic science research for pain is the fact that reflexive behaviors measured in many animal studies or acute pain thresholds measured in humans may focus more on percepts and responses to the presence of external threats rather than on the percepts and tonic pain caused by actual internal body injury that would better reflect the affective/emotional experience of pain.^49^ Furthermore, many animal studies performed (eletro)acupuncture under anesthesia to eliminate stress in wake animals, although behaviors were evaluated in awake animals after completion of acupuncture. As such, mechanisms gained from animal studies need to be validated in human studies. Note that new acupuncture studies start to focus on not only the relief of pain symptoms but also to investigate modulation of the underlying pathophysiology, such as inflammation that drives the activation of pain pathways, which could lead to therapeutic efficacy.^80^

There are ongoing debates regarding the presence of acupoint specificity for acupuncture analgesia. Animal studies, performed under anesthetic conditions, do strongly support the presence of acupoint specificity, although acupoint specificity is a relative term, influenced by the nature of autonomic neural pathways impacted by acupuncture, as well as by stimulation parameters that include stimulus intensities, frequencies, and the course of (typically) electroacupuncture treatment. For example, pioneering studies done since late 1970s show that acupuncture stimulation at limb and abdominal areas could drive specific vagal and sympathetic reflexes, respectively, thereby modulating gastrointestinal motility in a body region-dependent manner.^43,66^ More recent studies further show that low-intensity electroacupuncture in the hindlimb area of mice, but not the abdominal area, could suppress systemic inflammation through the activation of the vagal–adrenal axis. These anti-inflammatory effects depended on adrenal chromaffin cells expressing the neuropeptide Y and PROKR2^Cre^-marked sensory neurons of dorsal root ganglia that selectively innervate fascial tissues in the limb area.^21,29,44,47,70,102^ By contrast, high-intensity electroacupuncture at both the abdomen and the limb area is able to activate splenic adrenergic neurons through the spinal-sympathetic axis.^73^ The latter stimulation might produce either anti- or proinflammatory effects because of disease state-dependent changes in adrenergic receptor profiles in splenic immune cells.^73^

Moreover, animal studies may also help provide a better understanding of why human clinical acupuncture studies face challenges in designing adequate sham acupuncture as placebo control interventions. If traditional acupuncture efficacy is mediated by specific patient sensations with needling (see above), involving the activation of sensory neurons innervating subcutaneous, muscle, and deep fascia tissues,^23,37,47^ it does face a redundancy issue, that is, the superficial skin epidermis and hair follicles also contain a dense neural network, whose activation can also modulate pain.^8,49^ As such, commonly used non–skin-penetrating sham controls with sharp needling perception (which visually simulate skin penetration) should activate the superficial neural network. Thus, sham acupuncture may not be physiologically inert and might foster a better analgesic response in comparison to more classical placebo effects evoked by ingesting a sugar pill (see below). It should also be noted that placebo effects evoked by both real and sham acupuncture in humans may impact the autonomic nervous system,^19^ and this overlap could contribute to the small differences noted between real and sham acupuncture for pain disorders in prior clinical studies.^78^ Ultimately, prior basic science research and our growing understanding of the physiology affected by needling suggests that the acupuncture research field needs more development in designing truly inert sham controls and/or the migration to more pragmatic clinical trial designs (see below).

## 5. Human physiological and translational research

Physiological effects in response to acupuncture have been widely noted in humans, both at the site of the needle and further away. Many of these physiological responses are relevant to the experience of pain for chronic pain patients. For instance, ultrasound has imaged fascia (connective tissue) deformation in response to acupuncture needle manipulation,^38^ and optical imaging techniques have found that blood flow is elevated locally where the needle is inserted, with graded increases when the needle is inserted deeper and manually stimulated,^65^ as is common for many clinical acupuncture techniques. Connection to the nervous system, and ultimately brain function, may result from needle insertion into and through deep and superficial layers of fascia.^37^ Stimulation of the acupuncture needle can also include electrical stimulation, as noted above, which leads to better pain relief compared with manual stimulation, for both evoked experimental pain^39^ and some chronic pain disorders.^40^ Brain response to acupuncture stimulation, using techniques such as functional magnetic resonance imaging, has found that needle stimulation can impact activity in many emotion and cognitive processing brain areas—not just brain areas that respond to touch,^30^ potentially contributing to acupuncture applicability to the relief of pain, which is a multidimensional experience. Positron emission tomography imaging studies have noted how acupuncture increases binding of endorphin receptors in emotion-processing areas of the brain,^24^ supporting extensive basic research on acupuncture modulation of the brain's own “endogenous” opioids. However, acupuncture is a complex intervention, and nonneedling effects should also be considered. Patients' belief in therapy and the rich patient–clinician relationship can also help acupuncture reduce pain through therapeutic alliance, which has recently been studied by imaging concurrent brain activity in patients and acupuncturists to assess brain-to-brain concordance with hyperscanning functional magnetic resonance imaging.^2,14,15^ A growing number of studies have now incorporated brain imaging into longitudinal clinical trials of acupuncture, reinforcing the role of brain plasticity in pain reduction by acupuncture. Specifically, electroacupuncture was shown to improve somatotopic integrity for cortical representations in primary somatosensory cortex in persons experiencing carpal tunnel syndrome.^54^ Also, noninvasive MRI techniques, such as magnetic resonance spectroscopy, have been used to show that acupuncture can modulate excitatory (ie, glutamate) and/or inhibitory (ie, Gamma-Aminobutyric Acid, GABA) neurotransmitters in important pain processing regions of the brain, such as the insula cortex.^55^ These studies suggest that acupuncture is a specific form of a growing class of neuromodulation therapies,^59^ targeting peripheral nerves, with clear effects on the brain, ultimately reducing pain intensity and interference.

## 6. Clinical research and integration

Numerous randomized controlled trials (RCTs) of acupuncture for different chronic and/or cancer pain conditions have been published. A pooled individual subject meta-analysis (ie, mega-analysis) included data from more than 20,000 patients experiencing nonspecific musculoskeletal pain, osteoarthritis, chronic headache, or shoulder pain. This analysis showed that real acupuncture was superior to both sham acupuncture and nonacupuncture controls, although the difference between real acupuncture and sham acupuncture was small,^78^ as discussed above. In addition, a recent multicenter RCT study compared 20 sessions of manual acupuncture vs sham acupuncture and usual care for prophylaxis of episodic migraine without aura and reported superior outcomes for manual acupuncture compared with both control groups, suggesting that manual acupuncture can be an option for patients reluctant to use prophylactic drugs or when prophylactic drugs are ineffective.^93^ Another recent multicenter RCT comparing 12 weeks of real vs sham acupuncture or waitlist control in the treatment of aromatase inhibitor–related joint pain showed that there was a statistically significant decrease in joint pain following real acupuncture with long-term benefits that persisted at 40 weeks after discontinuation.^26^ Interestingly, in many studies, sham acupuncture has been shown to be more effective than a placebo pill for pain, suggesting that more research on specific acupuncture mechanisms is needed to better inform the design of placebo controls for acupuncture.^35,78^ For instance, an important variable across different acupuncture controls involves skin penetration, which has been found to provide better pain relief than nonpenetrating sham needles or placebo controls without needles.^52^

Acupuncture studies have shown that pain relief can occur within 30 minutes,^91^ whereas some relief can persist for months and even a full year following therapy.^26,53^ This suggests that acupuncture can sometimes address the root of the disease that drives pain, such as modulation of chronic inflammation. Thus, an extensive clinical trial literature supports the conclusion that acupuncture should be considered as an effective alternative or integrative nonpharmacological intervention for pain.

## 7. Clinical implication

The acceleration of clinical acupuncture research for pain control, as seen in the past decade, may stem from the growing appreciation of nonpharmacological therapies for pain management following the challenges of the opioid epidemic and the search for pain modalities to reduce opioid use.^32^ Numerous RCTs of acupuncture for different pain conditions have been published and have contributed to Clinical Recommendations and Guidelines for moderate to severe cancer pain,^18^ nonpharmacological prevention and treatment of chemotherapy-induced peripheral neuropathy,^36^ and an updated white paper on comprehensive acute pain care^62^ (Table 1). Because the quality of most available evidence of acupuncture therapy is weak to moderate, improving research design and increasing study sample size are needed for the evidence base supporting acupuncture analgesia. To accomplish this goal, a recent consensus by an international panel to promote high-quality acupuncture trials has been released.^100^

**Table 1:** Summarized acupuncture-related publications for pain control presented as clinical practice guideline/clinical recommendations with limited quality and homogeneity of methodology.

Types of pain	Pain condition	Types of publication	Summary	References
1. Cancer-related pain	Moderate to severe cancer pain	Evidence-based clinical practice guideline	The guideline is contributed by medical oncologists, Chinese medicine/acupuncture practitioner, and 2 patients. Although the need for additional research is recommended, 3 recommendations by the grading of reccommendations assessment, development, and evaluation (GRADE) approach are proposed. (1) A strong recommendation for the treatment with acupuncture rather than without acupuncture to relieve pain in patients with moderate to severe cancer pain. (2) A weak recommendation for combining acupuncture/acupressure with other treatments to improve pain control and reduce opioid dosage and opioid-related side effects in patients with moderate to severe cancer pain who are treated with analgesics. (3) A strong recommendation for acupuncture treatment to relieve aromatase inhibitor–induced arthralgia in breast cancer patients.	Ge L, et al. *Chin Med* 2022;17:8.^18^
2. Cancer-related pain and neuropathic pain	Prevention and treatment of chemotherapy-induced peripheral neuropathy (CIPN)	Clinical recommendations of nonpharmacological interventions from a systematic scope review and an expert consensus process	Acupuncture/acupressure is 1 of the 13 nonpharmacological interventions identified by the best evidence on complimentary treatments for CIPN. Efficacy of acupuncture on CIPN is controversial. The choice to select this treatment should be in consideration of the patient's perspective and the individual clinical expertise of the health professionals	Klafke N, et al. *Med Sci* 2023;11:15.^36^
3. Acute pain	Comprehensive acute pain care	The Academic Consortium Pain Task Force White Paper Update	22 systematic reviews and 17 meta-analyses Most reviews find acupuncture therapy to be effective and safe for acute pain, with the potential to avoid or reduce opioid dependence. There are suggestions to include acupuncture in comprehensive acute pain care and consider future multicenter trials to clarify the dosage and generalizability of acupuncture for acute pain in the emergency department	Nielsen A, et al. *Pain Med* 2022;23:1582–612.^62^

We should also note that although acupuncture as monotherapy has been reported to be effective in some pain conditions, recent evidence has been shown that different pain-related clinical targets and complementary interventions, such as patient-controlled analgesia,^11^ analgesics ladder pain program,^42^ rehabilitation training,^98^ and moxibustion and cupping,^104^ integrate with acupuncture to produce better clinical outcomes by reducing the dosage of analgesic used.

## 8. Limitations of the key studies or references employed

Several limitations to our approach in summarizing acupuncture research and clinical applicability should be noted. Of importance is the main limitation that this article is a narrative review. There was no systematic search or screening, no systematic quality appraisal of included studies, and a solely narrative approach to synthesize the evidence. In addition, the philosophical underpinnings of acupuncture therapy, as part of traditional East Asian medicine, has an extensive history and we have only touched on this background here. A more thorough historical description of acupuncture therapy can be found elsewhere.^17,34,48^ However, throughout the article, we point out the opportunities for future scientific research to fill in important knowledge gaps, enhancing our understanding of acupuncture analgesia.

## 9. Conclusion

Progress in acupuncture preclinical basic science research along with better integration of human basic science research with clinical longitudinal trials for pain control promises to improve the already significant evidence base supporting acupuncture analgesia. Because of the low-risk profile under standardized techniques and potential of cost-effectiveness, acupuncture should be considered a practical, nonpharmacological, integrative intervention for acute and chronic pain management.

## Disclosures

The authors have no conflicts of interest to declare.

S.N. is professor of Anesthesiology at Faculty of Medicine, Chulalongkorn University, and King Chulalongkorn Memorial Hospital, a committee member of the Thai Sub-board of Anesthesiology in Pain Medicine since 2009, and an International Association for the Study of Pain councilor (2020–2026). She is also the past president of the Thai Association for the Study of Pain for 2 consecutive terms (2014–2018). She was trained as a medical acupuncturist in the training program of Traditional Chinese Medicine hosted by the Ministry of Public Health of Thailand and in collaboration with Chengdu University of Traditional Chinese Medicine, Sichuan, the People's Republic of China.

V.N. is Professor of Physical Medicine and Rehabilitation, as well as Radiology, at Harvard Medical School. He is also the director of the Scott Schoen and Nancy Adams Discovery Center for Recovery from Chronic Pain at Spaulding Rehabilitation Hospital and the Center for Integrative Pain Neuroimaging (CiPNI) at the Martinos Center for Biomedical Imaging at Massachusetts General Hospital. He has been a pain neuroimaging researcher for more than 20 years and has more than 250 publications in leading peer-reviewed scientific journals. He is past president of the Society for Acupuncture Research and serves on the board of the US Association for the Study of Pain (USASP) and in numerous conference, journal, and National Institute of Health review panels.

Q.M. is professor of Neurobiology and the director of Bioelectronic Medicine at Westlake University, China. He has been studying the somatosensory system for nearly 3 decades, first on sensory neuron development, then on spinal circuit mapping, and more recently on the neuroanatomical basis of acupuncture in modulating inflammation and pain. He was professor of Neurobiology at Harvard Medical School before he moved to Westlake University in 2022.

## Appendix A. Supplemental digital content

Supplemental digital content associated with this article can be found online at http://links.lww.com/PR9/A244.
